# Etiologies and Diagnostic Yield of Bone Marrow Evaluation in Adults Living with HIV in Venezuela: A Cross-Sectional Study

**DOI:** 10.3390/v18090992

**Published:** 2026-09-09

**Authors:** Lily M. Soto-Avila, Higinio Fernández-Sánchez, Alfonso J. Rodriguez-Morales

**Affiliations:** 1 Instituto de Medicina Tropical, Facultad de Medicina, Universidad Central de Venezuela, Caracas 1050, Venezuela; dralilysoto@gmail.com; 2 Cizik School of Nursing, The University of Texas Health Science Center at Houston, Houston, TX 77030, USA; higinio.fernandezsanchez@uth.tmc.edu; 3 Faculty of Health Sciences, Universidad Científica del Sur, Lima 15046, Peru

**Keywords:** HIV, AIDS, bone marrow aspiration, bone marrow biopsy, histoplasmosis, tuberculosis, opportunistic infections

## Abstract

**Background:** Bone marrow abnormalities in people living with HIV may reflect infectious, neoplastic, or inflammatory processes, particularly in advanced disease presenting with fever of unknown origin or unexplained cytopenias. In Latin America, contemporary data on the causes and diagnostic contribution of bone marrow evaluation remain limited, especially in resource-constrained settings. **Aim:** To characterize the etiologies, histopathological patterns, and diagnostic contribution of bone marrow evaluation in adults living with HIV at a tertiary referral center in Venezuela. **Methods:** We conducted a cross-sectional study with retrospective and prospective case ascertainment among adults with confirmed HIV infection who underwent bone marrow aspiration, biopsy, or both for suspected infiltrative disease between January 2019 and April 2024. We analyzed clinical, laboratory, histopathological, microbiological, and molecular data at the index bone marrow evaluation. Test-specific denominators are reported because diagnostic investigations were not uniformly available. **Results:** Forty-two patients were included (35 retrospectively and 7 prospectively); 59.5% were male and the median age was 39 years (IQR: 31.75–50.0). At presentation, 67% were newly diagnosed with HIV and 79% were ART-naive; the median CD4+ T-cell count was 98.5 cells/mm^3^. Histopathology was evaluable in 37/42 patients; 31/37 (83.8%) showed findings compatible with infectious involvement and 6/37 (16.2%) showed malignancy. *Histoplasma capsulatum* was isolated in 17/35 fungal cultures (48.6%), *Mycobacterium tuberculosis* in 13/33 mycobacterial cultures (39.4%), and CMV PCR on bone marrow aspirate was positive in 2/3 patients tested. The final etiologic classification was infectious in 36/42 patients (85.7%) and neoplastic in 6/42 (14.3%). **Conclusions:** In this highly selected cohort of adults with advanced HIV disease, infectious etiologies-particularly histoplasmosis and tuberculosis-predominated. Bone marrow aspiration and biopsy, interpreted together with microbiological and molecular testing, provided clinically relevant diagnostic information in patients with prolonged fever, cytopenias, or otherwise inconclusive investigations. The findings support earlier HIV diagnosis and ART initiation and improved access to fungal, mycobacterial, and molecular diagnostics in resource-limited settings.

## 1. Introduction

Bone marrow abnormalities are common in advanced Human Immunodeficiency Virus (HIV) disease and may result from opportunistic infections, hematologic malignancies, inflammatory processes, or treatment-related effects. Their clinical presentation is often nonspecific, with fever of unknown origin, cytopenias, weight loss, and hepatosplenomegaly overlapping across etiologies. In Latin America, approximately 2.5 million people are living with HIV [1]. Since the initial description of Acquired Immunodeficiency Syndrome (AIDS) [2,3], combination antiretroviral therapy (ART) has transformed the natural history of HIV infection and substantially reduced AIDS-defining illness and mortality [4]. Nevertheless, late HIV presentation and advanced immunosuppression remain important challenges in resource-constrained settings.

Bone marrow involvement may arise from infectious, neoplastic, or inflammatory processes [5]. Relevant infectious causes include *Mycobacterium tuberculosis*, *Histoplasma capsulatum*, cytomegalovirus (CMV), and Parvovirus B19, whereas neoplastic causes include HIV-associated lymphomas and leukemias [6,7]. Bone marrow aspiration and biopsy may be considered when noninvasive investigations do not establish a diagnosis [8,9]. Disseminated histoplasmosis is particularly important in Latin America and remains under-recognized where rapid fungal diagnostics are not routinely available [10,11,12], while viral infections such as CMV may also remain undiagnosed when molecular testing is limited [13]. HIV-associated malignancies remain clinically important [14,15], and improved survival with ART has increased the cumulative period during which malignancies may develop [16,17].

Venezuela provides a clinically relevant setting in which late presentation, interruptions in HIV care, and constrained access to specialized diagnostic testing may influence the spectrum of advanced HIV disease. Contemporary Venezuelan data on bone marrow pathology in people living with HIV are scarce. Studies from other settings, including broader cohorts of bone marrow granulomas in high-tuberculosis-burden environments, show that marrow morphology and etiologic patterns vary with patient selection, local pathogen epidemiology, and diagnostic methods [7]. Direct numerical comparisons across studies therefore require caution.

Accordingly, this study aimed to characterize the etiologic distribution, histopathological patterns, and clinical characteristics of adults living with HIV who underwent bone marrow evaluation at the University Hospital of Caracas, and to separately describe the contribution of histopathology, microbiological culture, and molecular testing to the final etiologic classification.

## 2. Methods

### 2.1. Study Design

We conducted a cross-sectional study with retrospective and prospective case ascertainment. The retrospective component included eligible patients identified from January 2019 through December 2022, whereas the prospective component included consecutive eligible patients evaluated from January 2023 through April 2024. For each participant, we analyzed clinical, immunologic, laboratory, microbiological, and histopathological variables at a single predefined index time point corresponding to the bone marrow (BM) aspiration (BMA) and/or biopsy. The study was not designed as a longitudinal cohort and did not evaluate incidence, time-to-event outcomes, or post-biopsy survival.

### 2.2. Setting

The study was conducted at the University Hospital of Caracas (Hospital Universitario de Caracas), a national tertiary-care referral center affiliated with the Central University of Venezuela, Caracas, Venezuela. The hospital provides internal medicine, hematology, and infectious diseases services and receives patients from Caracas and other regions of Venezuela. We obtained retrospective data through a systematic review of available paper-based and electronic medical records. We recorded prospective data during clinical care using the same standardized data collection form. The bone marrow evaluation served as the reference point for the cross-sectional analysis.

### 2.3. Participants and Sample Size

**Figure 1:**
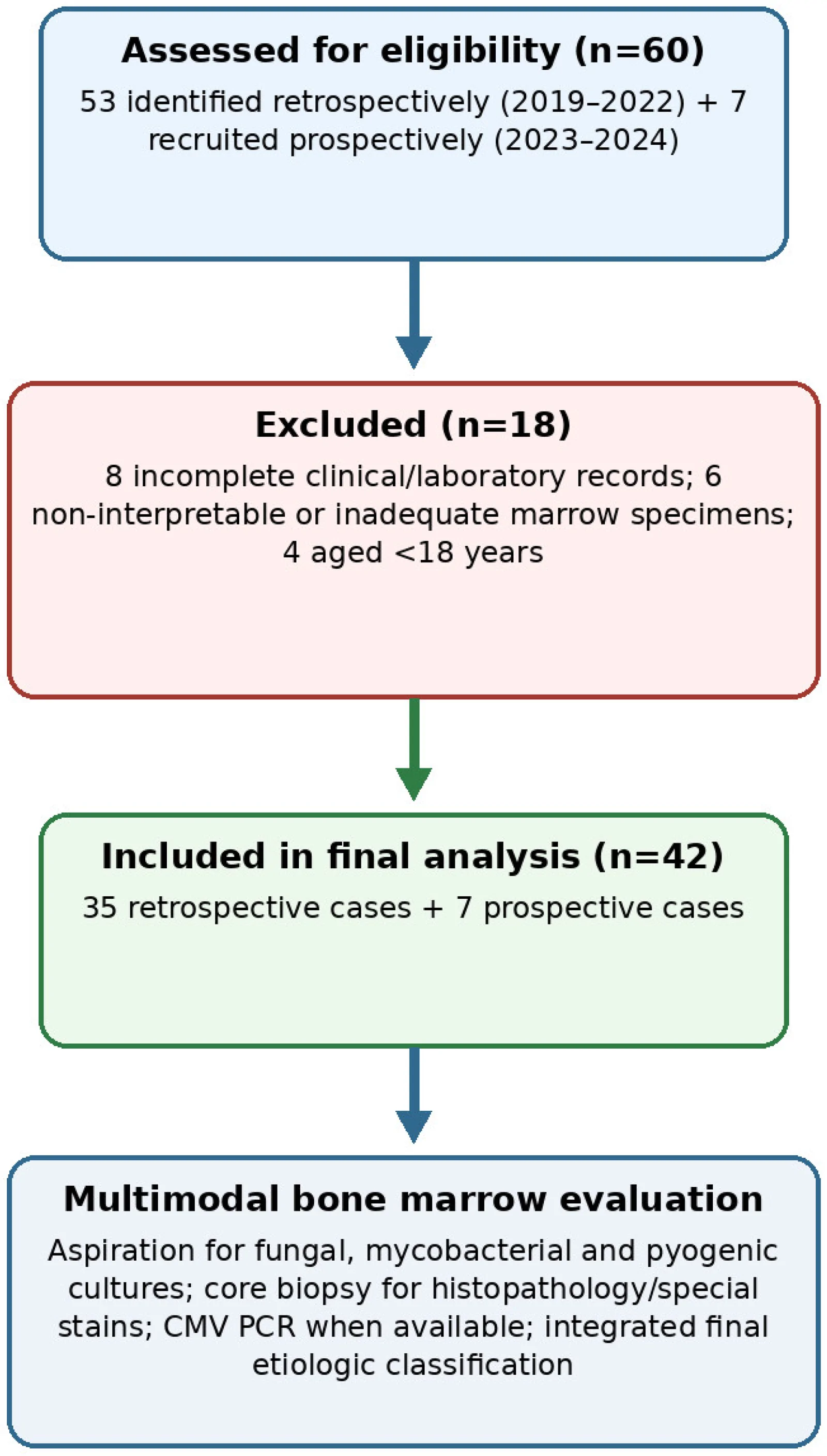
Patient enrollment and diagnostic flowchart.

Eligible participants were adults aged ≥18 years with confirmed HIV infection who underwent bone marrow aspiration, bone marrow biopsy, or both due to clinical suspicion of infiltrative bone marrow disease (Figure 1). Clinical suspicion was defined by the presence of one or more of the following:

- fever of unknown origin (≥38 °C for ≥2 weeks without identified cause),
- unexplained cytopenia affecting one or more hematopoietic lineages,
- hepatosplenomegaly, or
- clinical suspicion of hematologic malignancy.

These clinical criteria were established a priori and applied consistently to both retrospective and prospective case ascertainment, consistent with published clinical indications for bone marrow evaluation in advanced HIV disease [8,9].

For the retrospective component, we identified participants through a structured search of institutional medical records using predefined keywords (“HIV,” “AIDS,” “bone marrow,” “lymphoma,” “leukemia”), followed by manual eligibility verification. Two investigators independently verified eligibility and resolved discrepancies by consensus. For the prospective component, clinicians from the Internal Medicine, Hematology, and Infectious Diseases services recruited consecutive patients who met the inclusion criteria during evaluation. Patients were excluded if they:

- were younger than 18 years,
- did not have a bone marrow specimen available for analysis,
- had incomplete records lacking key variables (demographics, HIV status, or bone marrow results), or
- had samples that were not processed or yielded non-interpretable results. Non-interpretable specimens were defined as samples with insufficient cellularity, severe crush artifact, or inadequate tissue to permit reliable histopathological assessment, as determined by the reviewing pathologist.

Sixty patients were screened for eligibility: 53 were identified retrospectively (2019–2022), and 7 were recruited prospectively (2023–2024). Eighteen were excluded: 8 because of incomplete clinical/laboratory records, 6 because of non-interpretable or inadequate bone marrow specimens, and 4 because they were younger than 18 years. The final cohort therefore comprised 42 patients: 35 retrospective cases and 7 prospective cases. No formal sample size calculation was performed because the study was descriptive and included all eligible cases available during the study period.

### 2.4. Variables and Definitions

The primary outcome was the etiologic classification of infiltrative bone marrow disease. Etiologies were categorized as:

1. Infectious, based on identification of pathogens by culture, histopathology, or molecular methods
2. Neoplastic, based on histopathological diagnosis of malignancy
3. Non-specific/inconclusive, when no definitive etiology could be established

Histoplasmosis was considered confirmed when *Histoplasma capsulatum* was isolated from bone marrow culture and/or intracellular yeasts compatible with Histoplasma were directly visualized on marrow histology using appropriate fungal stains. Bone marrow tuberculosis was classified as confirmed when *Mycobacterium tuberculosis* was isolated from bone marrow mycobacterial culture and/or acid-fast bacilli were demonstrated on Ziehl–Neelsen staining of the marrow specimen. Probable bone marrow tuberculosis was defined as caseating/necrotizing or non-caseating granulomatous inflammation in a clinically compatible patient without direct microbiological confirmation, after absence or exclusion of fungal infection by available fungal stains and culture. We did not consider granulomatous inflammation alone to be confirmed tuberculosis. We defined histopathological findings compatible with infectious involvement as caseating or non-caseating granulomatous inflammation, granulomatous inflammation with marked hypocellularity/gelatinous transformation, and/or histiocytic proliferation containing intracellular microbial structures on routine hematoxylin and eosin staining or special stains. We treated these morphologic patterns as compatible with infectious involvement rather than pathogen-specific confirmation unless we directly demonstrated an organism or a marrow-derived microbiological/molecular test was positive. We analyzed routine pyogenic bacterial culture separately from specialized mycobacterial culture [18]. Collected variables included:

- Demographic variables: age, sex
- HIV-related variables: cluster of differentiation CD4+ T-cell count (cells/mm^3^), HIV viral load (copies/mL), time since diagnosis, and antiretroviral therapy (ART) status at presentation
- Clinical variables: fever, weight loss, constitutional symptoms, hepatosplenomegaly
- Laboratory variables: hemoglobin (g/dL), leukocyte count (cells/mm^3^), platelet count (cells/mm^3^), lactate dehydrogenase (LDH, IU/mL)
- Bone marrow findings: cellularity (hypocellular, normocellular, hypercellular), presence of granulomas, malignant infiltration
- Microbiological and molecular results: fungal culture, routine pyogenic bacterial culture, mycobacterial culture, and CMV Polymerase Chain Reaction (PCR) when available. HIV viral load and CD4+ T-cell count were measured in peripheral blood. When available, we performed CMV PCR on bone marrow aspirate. We did not use Xpert/GeneXpert testing for *Mycobacterium tuberculosis* in this cohort.

We operationally classified advanced HIV disease as a CD4+ T-cell count < 200 cells/mm^3^ for the analyses presented here, consistent with the immunologic threshold used throughout the study [19,20]. ART-naive status was defined as no prior exposure to antiretroviral therapy at the time of bone marrow evaluation. We prespecified final etiologic categories as infectious, neoplastic, or non-specific/inconclusive. We retained the non-specific/inconclusive category as a possible outcome, even though no patient ultimately remained in this category after integrating the available findings.

### 2.5. Data Sources and Measurement

We extracted retrospective data using a standardized abstraction form and reviewed them for consistency; a second investigator independently reviewed a random 20% subset of records. We recorded prospective cases using the same standardized form. We obtained bone marrow aspirates and core biopsies according to institutional protocols. Histopathological evaluation included hematoxylin and eosin staining and, when indicated, Ziehl–Neelsen, periodic acid-Schiff, and Gomori methenamine silver stains.

Experienced pathologists interpreted the biopsies according to institutional protocols; we did not perform formal double reading or assess interobserver agreement. We performed fungal cultures on Sabouraud dextrose agar, mycobacterial cultures on Lowenstein–Jensen medium, and routine pyogenic bacterial cultures on standard blood agar and MacConkey agar. We measured HIV viral load in peripheral blood. When available, we performed CMV PCR on bone marrow aspirate; Parvovirus B19 PCR was not available in this cohort. We performed histopathological and microbiological analyses at the University Hospital of Caracas, while molecular testing was done either locally or at external reference laboratories in Caracas. Because of resource limitations, not all patients underwent the same diagnostic panel.

### 2.6. Bias Assessment

Selection and referral bias are inherent because we included only patients with sufficiently severe or unexplained manifestations to undergo bone marrow evaluation. Retrospective cases were also susceptible to information bias from incomplete documentation. We used standardized abstraction forms and predefined variable definitions to improve consistency. Diagnostic misclassification was possible because access to microbiological and molecular tests was not uniform, and limited use of molecular testing may have led to under-recognition of viral or mixed infections. We handled missing data using complete-case analysis without imputation.

### 2.7. Quantitative Variables

We analyzed continuous variables as continuous measures or categories them using clinically relevant thresholds. These included:

- CD4+ T-cell count (<200 vs. ≥200 cells/mm^3^)
- Hemoglobin (anemia defined according to World Health Organization (WHO) criteria)
- LDH (>600 IU/mL as clinically relevant threshold)

We used categorization to facilitate clinical interpretation.

### 2.8. Statistical Methods

We performed statistical analyses using IBM SPSS Statistics version 26.0 (IBM Corp., Armonk, NY, USA). We summarized continuous variables as medians and interquartile ranges because the Shapiro–Wilk test showed most distributions were non-normal. We summarized categorical variables as frequencies and percentages. Exploratory subgroup comparisons used the Mann–Whitney U test for continuous variables and chi-square or Fisher’s exact test for categorical variables, as appropriate. A two-sided *p*-value < 0.05 was used as the nominal significance threshold. Analyses used complete cases, and we did not impute missing values. Because the sample was small and the study was not powered for subgroup comparisons, all comparative analyses were considered exploratory; non-significant findings were not interpreted as evidence of equivalence or similarity.

### 2.9. Ethical Considerations

The Bioethics Committee of the University Hospital of Caracas approved the study (Approval No. 40). Written informed consent was obtained from participants in the prospective component. For the retrospective component, the requirement for informed consent was waived because we used previously collected anonymized data. All data were anonymized before analysis, and study investigators had restricted access to the database.

The study complied with the Código de Deontología Médica of the Federación Médica Venezolana, the Normas de Buena Práctica Clínica established by the Venezuelan Ministry of Health, and applicable provisions of the Ley del Ejercicio de la Medicina. All procedures were performed in in accordance with national regulations and the ethical standards of the Declaration of Helsinki [21].

## 3. Results

### 3.1. Study Population and Baseline Characteristics

A total of 42 adults living with HIV were included: 35 were ascertained retrospectively and 7 prospectively. We assigned all 42 patients a final predominant etiologic category after integrating available marrow-derived histopathological, microbiological, and molecular findings with the clinical context; this final classification rate should not be interpreted as the diagnostic yield of any single modality. Most participants were male (25/42, 59.5%), and the median age was 39 years (IQR: 31.75–50.0). At presentation, 28/42 (66.7%) were newly diagnosed with HIV, and 33/42 (78.6%) were ART-naive. The higher proportion of ART-naive patients reflects inclusion of previously diagnosed individuals who had not initiated or no longer had access to ART, a pattern occurring in the context of documented disruptions in HIV care during the study period [22,23]. Denominators vary across analyses because diagnostic and laboratory testing was not uniformly available.

### 3.2. Indications for Bone Marrow Evaluation

The primary clinical indications for bone marrow aspiration and/or biopsy were fever of unknown origin and unexplained cytopenias involving one or more hematologic lineages. Among patients with complete hematologic data, pancytopenia occurred in 42.11% (16/38).

Four patients had incomplete hematologic panels at the time of evaluation because laboratory availability was limited during the study period; therefore, we assessed pancytopenia only in those with simultaneous hemoglobin, leukocyte, and platelet counts (*n* = 38). Other indications included suspected hematologic malignancy and the presence of systemic symptoms such as weight loss and hepatosplenomegaly. Among the 42 patients, fever of unknown origin was the primary indication in 71.43% (30/42), unexplained cytopenias in 64.29% (27/42), suspected hematologic malignancy in 19.05% (8/42), and hepatosplenomegaly in 45.24% (19/42); these indications were not mutually exclusive. These indications were consistent with the predefined criteria for suspected infiltrative bone marrow disease described in the Section 2.

### 3.3. Bone Marrow Histopathological Findings

Table 1 summarizes diagnostic test availability, positivity, and contribution to the final etiologic classification. Core biopsy histopathology was evaluable in 37/42 patients (88.1%). Among evaluable biopsies, 31/37 (83.8%) showed histopathological findings compatible with infectious involvement and 6/37 (16.2%) demonstrated malignant marrow infiltration. The most frequent morphologic patterns were hypocellularity and granulomatous inflammation (14/37, 37.8% each), with one additional biopsy showing both granulomatous inflammation and hypocellularity. These histopathological categories describe morphologic patterns and should not be interpreted as pathogen-specific confirmation without direct organism identification or a positive marrow-derived microbiological test.

### 3.4. Microbiological and Molecular Findings

Microbiological testing complemented histopathology and provided pathogen-specific evidence in a subset of patients. We isolated *Histoplasma capsulatum* from bone marrow culture in 17/35 patients tested (48.6%), and *Mycobacterium tuberculosis* in 13/33 mycobacterial cultures (39.4%). Routine pyogenic bacterial cultures were performed in 35 patients and were all negative. We performed CMV PCR on bone marrow aspirate in 3 patients, and it was positive in 2/3 (66.7%). Parvovirus B19 PCR was not performed because of limited molecular diagnostic availability. In the final integrated etiologic classification, 36/42 patients (85.7%) were classified as having an infectious etiology and 6/42 (14.3%) as having a neoplastic etiology. No patient remained in the prespecified non-specific/inconclusive category.

### 3.5. Hematologic and Biochemical Characteristics

Patients had substantial hematologic abnormalities. The median leukocyte count was 3320 cells/mm^3^ (IQR: 2177.5–6005), hemoglobin 8.25 g/dL (IQR: 6.65–9.60), and platelet count 96,500 cells/mm^3^ (IQR: 39,500–194,250). The median CD4+ T-cell count was 98.5 cells/mm^3^ (IQR: 48.25–120.5; available in 38/42 patients), and the median HIV viral load was 119,100 RNA copies/mL (IQR: 1310–550,000; available in 34/42). The median LDH was 761 IU/mL (IQR: 376.75–2008.5); 19/36 patients with available LDH values (52.8%) had levels > 600 IU/mL. Six patients lacked LDH measurements because the assay was not available at the time of evaluation.

### 3.6. Exploratory Comparative Analyses

Exploratory subgroup analyses compared patients by malignancy status, histoplasmosis status, and tuberculosis status using age, CD4+ T-cell count, HIV viral load, leukocyte count, hemoglobin, hematocrit, platelet count, and LDH. We found no statistically detectable differences in the evaluated continuous variables (all *p*-values > 0.05) and no statistically significant associations for the categorical clinical variables. Given the small sample size and multiple exploratory comparisons, these findings should not be interpreted as evidence of equivalence between etiologic groups.

### 3.7. Antiretroviral Therapy Exposure

At the index bone marrow evaluation, 33/42 patients (78.6%) were ART-naive and 9/42 (21.4%) were receiving ART. Regimens among previously treated patients were heterogeneous, reflecting differences in timing of HIV diagnosis and treatment access during the study period.

Venezuela experienced major ART supply disruptions during the years preceding the study [22,24]. In this selected cohort, ART-naive status, advanced immunosuppression, and severe disseminated disease co-occurred; these observations should not be interpreted as establishing that treatment interruption caused the distribution of specific opportunistic pathogens. Because of the small sample size and heterogeneous treatment histories, we did not perform stratified analyses by ART regimen.

## 4. Discussion

This study provides contemporary data on adults living with HIV who underwent bone marrow evaluation for suspected infiltrative disease at a tertiary referral center in Venezuela. The cohort was characterized by advanced immunosuppression, a high proportion of ART-naive patients, and a predominance of infectious etiologies. These features are consistent with a highly selected population referred for invasive diagnostic evaluation and should not be generalized to all people living with HIV in Venezuela. The demographic profile, including a median age of 39 years and male predominance, is broadly consistent with epidemiologic patterns and other bone marrow series in people living with HIV [25,26].

The high frequency of newly diagnosed and ART-naive patients is clinically important because it illustrates the burden of late presentation within this referral cohort. Similar studies have evaluated marrow abnormalities in patients with advanced HIV and unexplained cytopenias or fever, but comparisons should account for differences in patient selection, disease severity, geographic pathogen endemicity, and diagnostic infrastructure [27,28]. In Venezuela, prolonged disruptions in health services and ART availability provide important context for the high proportion of untreated patients observed here [22,23]. These co-occurring factors should not be interpreted as demonstrating a causal relationship with the distribution of specific marrow pathogens.

Granulomatous inflammation and hypocellular marrow were frequent histopathological patterns in this cohort. Granulomas are not specific for tuberculosis and may be associated with fungal, mycobacterial, and other infectious or inflammatory conditions; their interpretation therefore requires microbiological and clinical correlation. Wang et al. described bone marrow granulomas in a broader high-tuberculosis-burden setting [7], whereas HIV-specific series have reported a wider range of marrow abnormalities, including hypocellularity and opportunistic infections [26,29]. The present findings reinforce the limited specificity of marrow morphology alone and the importance of integrating histopathology with culture, special stains, and molecular testing when available. In Latin America, disseminated histoplasmosis remains underdiagnosed, particularly where antigen testing is unavailable [11,30,31].

Fever of unknown origin and unexplained cytopenias were the principal indications for bone marrow evaluation. In this setting, marrow examination can provide clinically relevant information when noninvasive investigations are inconclusive or unavailable, but the present study did not measure time to diagnosis or compare marrow examination with alternative diagnostic pathways. LDH was frequently elevated, with a median value of 761 IU/mL. Although LDH > 600 IU/mL is used locally as a supportive clue for disseminated histoplasmosis and has been described in previous reports [32], LDH is nonspecific and may also be elevated in malignancy, hemolysis, and other systemic illnesses.

The exploratory subgroup analyses did not identify statistically significant differences among patients with histoplasmosis, tuberculosis, or malignancy. Because the sample was small and these analyses were underpowered, do not interpret non-significant *p*-values as demonstrating similarity or equivalence between groups. Rather, the observed clinical and laboratory overlap illustrates why pathogen-specific microbiological evidence and definitive histopathology remain important when differentiating opportunistic infection from malignancy.

Infectious etiologies accounted for 36/42 (85.7%) of the final etiologic classifications, with histoplasmosis and tuberculosis predominating. This distribution is compatible with the epidemiology of advanced HIV disease in Latin America, where disseminated histoplasmosis remains underdiagnosed and may rival tuberculosis as a major opportunistic infection [30,31]. Importantly, the percentages of infectious and neoplastic etiologies describe the final etiologic distribution; they are not measures of the diagnostic yield of a single bone marrow procedure. For tuberculosis specifically, we distinguished culture-confirmed disease from probable marrow involvement based on compatible granulomatous histology without direct microbiological confirmation. Bone marrow tuberculosis is a severe extrapulmonary manifestation and may present with granulomatous marrow involvement [33].

Cytopenias across multiple lineages were common, including a high frequency of thrombocytopenia. Differences in hematologic patterns among published studies may reflect variation in case mix, disease stage, and local epidemiology rather than a uniform HIV-associated phenotype. In selected patients with advanced HIV disease, persistent fever, cytopenias, hepatosplenomegaly, or otherwise unexplained systemic illness, bone marrow aspiration and biopsy can contribute useful diagnostic information. Published diagnostic-yield estimates vary substantially across selected HIV cohorts [8,34], underscoring the importance of reporting the contribution of aspiration, core biopsy, culture, and molecular testing separately rather than summarizing them as a single estimate.

### 4.1. Implications for Policy, Practice, and Research

The findings have practical implications for care in resource-limited settings. Earlier HIV diagnosis and timely linkage to ART remain central to reducing advanced disease presentations. For patients who nevertheless present with severe cytopenias, prolonged fever, or suspected disseminated infection or malignancy, access to bone marrow aspiration and biopsy should be accompanied by adequate microbiological support, including fungal and mycobacterial culture and, where feasible, rapid fungal antigen and molecular testing. Other opportunistic infections that may involve bone marrow in Latin America, including visceral leishmaniasis, should also remain in the differential diagnosis [35]. Future studies should be multicenter and prospective, use standardized diagnostic panels, and include longitudinal outcomes so that the diagnostic performance, incremental value, cost-effectiveness, co-infection burden, treatment response, and survival implications of bone marrow evaluation can be assessed more rigorously.

### 4.2. Limitations and Strengths

This study has several limitations. First, the sample size was small (*n* = 42), limiting statistical power for subgroup comparisons; therefore, interpret all comparative analyses as exploratory. Second, this was a single-center, biopsy-selected referral cohort, creating selection and referral bias and limiting generalizability to the broader population of people living with HIV. Third, the combination of retrospective and prospective case ascertainment introduced heterogeneity in data completeness, and the retrospective component was particularly susceptible to missing information. Fourth, diagnostic testing was not uniform: molecular assays, specialized cultures, and contemporary fungal diagnostics were unavailable for some patients, which may have led to under-recognition of viral, fungal, or other co-pathogens and mixed marrow infections. Assigning a single predominant etiology to each patient may also have obscured concurrent conditions. Fifth, no formal interobserver agreement assessment was performed for histopathology. Finally, longitudinal outcomes, including treatment response and post-biopsy survival, were not systematically collected. Strengths include the integration of clinical, histopathological, microbiological, and molecular information and the provision of contemporary real-world data from a Venezuelan tertiary-care setting where evidence on HIV-associated marrow disease remains limited.

## 5. Conclusions

In this highly selected cohort of adults living with advanced HIV disease at a tertiary referral center in Venezuela, infectious etiologies predominated, particularly histoplasmosis and tuberculosis, while hematologic malignancies accounted for the remaining final classifications. Clinical and laboratory features overlapped substantially across etiologies, underscoring the need for pathogen-specific microbiological testing and definitive histopathology whenever feasible. Bone marrow aspiration and core biopsy provided clinically relevant information in patients with prolonged fever, cytopenias, and otherwise inconclusive investigations, but each diagnostic modality should be interpreted separately. The 85.7% infectious and 14.3% neoplastic figures describe the final etiologic distribution rather than the diagnostic yield of bone marrow examination itself. The high proportion of newly diagnosed and ART-naive patients further highlights the importance of earlier HIV detection and timely ART access. Expanded availability of fungal, mycobacterial, and molecular diagnostics, together with larger prospective studies incorporating standardized testing and longitudinal outcomes, is needed to refine the role of bone marrow evaluation in advanced HIV disease. Bone marrow examination also remains relevant when hematologic malignancy is suspected, including HIV-associated Hodgkin lymphoma [36].

## Figures and Tables

**Table 1:** Availability, positivity, and contribution of bone marrow diagnostic modalities among 42 adults living with HIV evaluated for suspected infiltrative bone marrow disease.

Diagnostic Modality/ Specimen	Available/Tested, *n*/*N* (%)	Positive or Abnormal, *n* (% of Tested)	Contribution to Final Etiologic Classification, *n*/*N* (%)
Core biopsy: histopathology (any abnormal finding)	37/42 (88.1)	37/37 (100.0)	37/42 (88.1)
Core biopsy: histopathology compatible with malignancy	37/42 (88.1)	6/37 (16.2)	6/42 (14.3)
Core biopsy: histopathological findings compatible with infectious involvement	37/42 (88.1)	31/37 (83.8)	31/42 (73.8)
Bone marrow aspirate: fungal culture for *Histoplasma capsulatum*	35/42 (83.3)	17/35 (48.6)	17/42 (40.5)
Bone marrow aspirate: mycobacterial culture for *Mycobacterium tuberculosis*	33/42 (78.6)	13/33 (39.4)	13/42 (31.0)
Bone marrow aspirate: routine pyogenic bacterial culture	35/42 (83.3)	0/35 (0.0)	0/42 (0.0)
Bone marrow aspirate: CMV PCR	3/42 (7.1)	2/3 (66.7)	2/42 (4.8)
Integrated final etiologic classification: combined marrow findings + clinical context	42/42 (100.0)	42/42 classified (100.0)	42/42 (100.0) *

Abbreviations: CMV, cytomegalovirus; PCR, polymerase chain reaction. Core biopsy was used for histopathology; bone marrow aspirate was used for fungal, mycobacterial, and routine pyogenic bacterial cultures and, when available, for CMV PCR. Percentages in the “Available/tested” and “Contribution” columns use the total study population (*N* = 42) as the denominator; percentages in the “Positive or abnormal” column use the number tested. “Histopathological findings compatible with infectious involvement” include granulomatous inflammation, granulomatous inflammation with marked hypocellularity/gelatinous transformation, or histiocytic proliferation with intracellular microbial structures. These patterns were not considered pathogen-specific confirmation unless supported by direct organism visualization or a positive marrow-derived microbiological or molecular test. * The integrated final classification rate is not the sensitivity or diagnostic yield of any single marrow procedure.

## Data Availability

The data presented in this study are available on reasonable request from the corresponding author due to privacy restrictions.

## References

[1] UNAIDS Latin America—2024 Global AIDS Update: The Urgency of Now. https://www.unaids.org/sites/default/files/media_asset/2024-unaids-global-aids-update-latin-america_en.pdf.

[2] Fee E., Brown T.M., Michael S. (2006). Gottlieb and the identification of AIDS. Am. J. Public Health.

[3] Bento D., Nguyen A.D. (2026). HIV-1-associated opportunistic infections. StatPearls.

[4] Tchounga B., Ekouevi D.K., Balestre E., Dabis F. (2016). Mortality and survival patterns of people living with HIV-2. Curr. Opin. HIV AIDS.

[5] Ji Y., Lu H. (2017). Malignancies in HIV-infected and AIDS patients. Adv. Exp. Med. Biol..

[6] Bisetegn H., Ebrahim H. (2021). The prevalence of thrombocytopenia and leucopenia among people living with HIV/AIDS in Ethiopia: A systematic review and meta-analysis. PLoS ONE.

[7] Wang Y., Tang X., Yuan J., Wu S., Chen G., Zhang M., Wang M., Zhang W., He J. (2018). Bone marrow granulomas in a high tuberculosis prevalence setting. Medicine.

[8] Hajiabdolbaghi M., Ataeinia B., Ghadimi F., SeyedAlinaghi S., Badie B.M., Dadras O., Rasoolinejad M. (2021). Bone marrow aspiration/biopsy in the evaluation of fever of unknown origin in patients with AIDS. Infect. Disord. Drug Targets.

[9] GeSIDA, División de Control de VIH, ITS, Hepatitis Virales y Tuberculosis (2023). Documento de Consenso de GeSIDA/División de Control de VIH, ITS, Hepatitis Virales y Tuberculosis del Ministerio de Sanidad respecto al Tratamiento Antirretroviral en Adultos Infectados por el Virus de la Inmunodeficiencia Humana (Actualización Enero 2023).

[10] Pasqualotto A.C., Lana D.D., Godoy C.S.M., Leitão T.D.M.J.S., Bay M.B., Damasceno L.S., Soares R.B.A., Kist R., Silva L.R., Wiltgen D. (2023). Single high dose of liposomal amphotericin B in human immunodeficiency virus/AIDS-related disseminated histoplasmosis: A randomized trial. Clin. Infect. Dis..

[11] Gómez B.L. (2019). Histoplasmosis: Desafíos diagnósticos. Rev. Méd. Univ. Antioq..

[12] Centers for Disease Control and Prevention Facts and Stats About Histoplasmosis. https://www.cdc.gov/histoplasmosis/php/statistics/index.html.

[13] Perello R., Vergara A., Monclus E., Jimenez S., Montero M., Saubi N., Moreno A., Eto Y., Inciarte A., Mallolas J. (2019). Cytomegalovirus infection in HIV-infected patients in the era of combination antiretroviral therapy. BMC Infect. Dis..

[14] Álvarez-Guevara D., Cuervo-Maldonado S., Sánchez R., Gómez-Rincón J., Ramírez N. (2017). Prevalence of defining malignancies in adult patients with HIV/AIDS in the National Cancer Institute of Colombia, 2007–2014. Rev. Fac. Med..

[15] Meijide H., Mena A., Pernas B., Castro A., López S., Vázquez P., Bello L., Baliñas J., Rodríguez-Martínez G., Pedreira J.D. (2013). Neoplasias en pacientes con infección por VIH: Estudio descriptivo de 129 casos en el período 1993–2010. Rev. Chil. Infectol..

[16] Trickey A., May M.T., Vehreschild J.-J., Obel N., Gill M.J., Crane H.M., Boesecke C., Hellkvist A., Samji H., Cavassini M. (2017). Survival of HIV-positive patients starting antiretroviral therapy between 1996 and 2013: A collaborative analysis of cohort studies. Lancet HIV.

[17] García-Abellán J., Del Río L., García J.A., Padilla S., Vivancos M.J., Del Romero J., Asensi V., Hernando A., García-Fraile L., Masiá M. (2019). Risk of cancer in HIV-infected patients in Spain, 2004–2015: The CoRIS cohort study. Enferm. Infecc. Microbiol. Clin..

[18] Guarner J., Brandt M.E. (2011). Histopathologic diagnosis of fungal infections in the 21st century. Clin. Microbiol. Rev..

[19] World Health Organization (2017). Guidelines for Managing Advanced HIV Disease and Rapid Initiation of Antiretroviral Therapy.

[20] World Health Organization (2025). WHO Guidelines on the Management of Advanced HIV Disease.

[21] World Medical Association (2013). World Medical Association Declaration of Helsinki: Ethical principles for medical research involving human subjects. JAMA.

[22] Page K.R., Doocy S., Reyna Ganteaume F., Castro J.S., Spiegel P., Beyrer C. (2019). Venezuela’s public health crisis: A regional emergency. Lancet.

[23] Pan Y., Horigian V.E., Saavedra J., Alonso E., Liao X., Botero V., Rodriguez A.E., Feaster D.J. (2025). HIV treatment disruption and viral suppression among Venezuelan immigrants living with HIV in Miami. AIDS Behav..

[24] PANCAP Antiretroviral Shortage Reaches 100% in Venezuela. https://pancap.org/pancap-releases/antiretroviral-shortage-reaches-100-in-venezuela/.

[25] Centers for Disease Control and Prevention HIV and Gay and Bisexual Men. https://www.cdc.gov/vitalsigns/hivgaybimen/index.html.

[26] Antinori S., Calattini S., Longhi E., Bestetti G., Piolini R., Magni C., Orlando G., Gramiccia M., Acquaviva V., Foschi A. (2007). Clinical use of polymerase chain reaction performed on peripheral blood and bone marrow samples for the diagnosis and monitoring of visceral leishmaniasis in HIV-infected and HIV-uninfected patients: A single-center, 8-year experience in Italy and review of the literature. Clin. Infect. Dis..

[27] Giraldo-Bahamón G., Ramírez-Ramos C.F., Motta-Quimbaya O.Y., Rivera-Marín J.D., Areiza-Paramo J.D., Serrano-Valderrama S.R., Vargas-Plazas H.I., Salinas-Cortés D.F. (2019). Evaluación de la médula ósea en pacientes citopénicos infectados con el virus de la inmunodeficiencia humana tipo 1 en hospital colombiano del tercer nivel. CES Med..

[28] Cáceres D.H., Gómez B.L., Restrepo Á., Tobón Á.M. (2012). Histoplasmosis y sida: Factores de riesgo clínicos y de laboratorio asociados al pronóstico de la enfermedad. Infectio.

[29] Dhawle M., Rathi V., Hiwale B. (2025). Study of bone marrow findings in human immunodeficiency virus (HIV)-positive patients in a tertiary care hospital. Cardiovasc. Hematol. Disord. Drug Targets.

[30] Nacher M., Adenis A., Mc Donald S., Do Socorro Mendonca Gomes M., Singh S., Lopes Lima I., Malcher Leite R., Hermelijn S., Wongsokarijo M., Van Eer M. (2013). Disseminated histoplasmosis in HIV-infected patients in South America: A neglected killer continues on its rampage. PLoS Negl. Trop. Dis..

[31] Adenis A.A., Valdes A., Cropet C., McCotter O.Z., Derado G., Couppié P., Chiller T., Nacher M. (2018). Burden of HIV-associated histoplasmosis compared with tuberculosis in Latin America: A modelling study. Lancet Infect. Dis..

[32] Carballo M., Guevara Palermo R.N., Aché A. (2005). Disseminated histoplasmosis in patients with acquired immunodeficiency syndrome: Usefulness of lactate dehydrogenase in its diagnosis. Vitae.

[33] Flores V., Trullas M.F., Jajati M., Sívori M. (2024). Tuberculosis extrapulmonar: Presentación atípica de compromiso de la médula ósea. Rev. Am. Med. Respir..

[34] Noiperm P., Saelue P. (2024). Predictive model for diagnostic yield of bone marrow examination in HIV-infected patients with fever of unknown origin. AIDS.

[35] Lindoso J.A.L., Moreira C.H.V., Cunha M.A., Queiroz I.T. (2018). Visceral leishmaniasis and HIV coinfection: Current perspectives. HIV AIDS (Auckl.).

[36] Gupta R.K., Marks M., Edwards S.G., Smith K., Fletcher K., Lee S.M., Ramsay A., Copas A.J., Miller R.F. (2014). A declining CD4 count and diagnosis of HIV-associated Hodgkin lymphoma: Do prior clinical symptoms and laboratory abnormalities aid diagnosis?. PLoS ONE.

